# Genetic diversity of Simao pine in China revealed by SRAP markers

**DOI:** 10.7717/peerj.6529

**Published:** 2019-02-26

**Authors:** Dawei Wang, Bingqi Shen, Hede Gong

**Affiliations:** 1 Key Laboratory for Forest Resource Conservation and Utilization in the Southwest Mountains of China, Ministry of Education, Southwest Forestry University, Kunming, Yunnan, China; 2 Key Laboratory for Forest Genetic and Tree Improvement & Propagation in Universities of Yunnan Province, Southwest Forestry University, Kunming, Yunnan, China; 3 School of Geography, Southwest Forestry University, Kunming, Yunnan, China

**Keywords:** Pinus kesiya Royle ex Gordon var. langbianensis, Genetic variation, SRAP markers, Conservation implications

## Abstract

**Background:**

Simao pine (*Pinus kesiya* Royle ex Gordon var. *langbianensis* (A. Chev.) Gaussen) is one of the most important tree species in the production of timber and resin in China. However, the genetic diversity of the natural populations has not been assessed to date. In this study, sequence related amplified polymorphism (SRAP) markers were used to investigate the genetic composition of natural Simao pine populations.

**Method:**

The SRAP markers were applied and their efficiency was compared using various statistical multivariate methods, including analysis molecular of variance (AMOVA), the unweighted pair group method with arithmetic mean (UPGMA), and Principal coordinate analysis (PCoA).

**Results:**

The 11 populations revealed a high level of genetic diversity (PPB = 95.45%, H = 0.4567, I = 0.6484) at the species level. A moderately low level of genetic differentiation (*G_st_* = 0.1701), and a slightly high level of gene flow (*N_m_* = 2.4403) were observed among populations using AMOVA. Eleven populations of Simao pine were gathered into four distinct clusters based on molecular data, and the results of UPGMA and PCoA also illustrated that assignment of populations is not completely consistent with geographic origin. The Mantel test revealed there was no significant correlation between geographic and genetic distance (*r* = 0.241, *p* = 0.090).

**Discussion:**

The SRAP markers were very effective in the assessment of genetic diversity in Simao pine. Simao pine populations display high levels of genetic diversity and low or moderate levels of genetic differentiation due to frequent gene exchange among populations. The low genetic differentiation among populations implied that conservation efforts should aim to preserve all remaining natural populations of this species. The information derived from this study is useful when identifying populations and categorizing their population origins, making possible the design of long term management program such as genetic improvement by selective breeding.

## Introduction

Simao pine (*Pinus kesiya* Royle ex Gordon var. *langbianensis* (A. Chev.) Gaussen), a geographic variant of *Pinus kesiya*, is distributed naturally in the humid and sub-humid areas of the tropical and subtropical zone within Yunnan Province (Wang et al., 2012a; Wang et al., 2012b). The species is one of the most important timber and resin production trees in China (Chen, Zhao & Wang, 2002; Cai et al., 2017; Zhu et al., 2017; Wang et al., 2018), and also has ecological and social benefits, in addition to economic ones (Ou et al., 2016; Li et al., 2018a; Li et al., 2018b). There is high potential for over utilization of the tree species and Simao pine breeding programs are constrained by a narrow genetic background and overexploitation (Zhao et al., 2016). Genetic improvement will be salient to future Simao pine breeding and in order to selectively breed Simao pine in a coordinated breeding scheme, genetic diversity analysis needs to be implemented.

Sequence-related amplified polymorphism (SRAP) is a kind of molecular marker technology based on polymerase chain reaction (PCR). The method is convenient, exhibits high co-dominance, is uncomplicated in the separation of strips and sequencing, and doesn’t require knowledge of the sequence information of species in advance (Li & Quiros, 2001). The SRAP markers are simple, reliable, easily detected, genome specific, highly polymorphic and commonly used in genomic applications (Ma et al., 2015). This form of analysis is also very effective for the assessment of genetic diversity (Shaye et al., 2018). For these reasons, SRAP markers are widely used in population genetic analysis of various plant species (Liu et al., 2016; Bhatt et al., 2017; Li et al., 2018a; Li et al., 2018b).

In this study, SRAP markers were used to investigate the genetic composition of natural Simao pine populations, which are limitedly distributed in Southwest Yunnan, China. The study’s aims were to evaluate genetic diversity at population and species levels in the Simao pine; assess the distribution of the genetic variation within and among populations, and construct a dendrogram demonstrating the genetic relationships among them. The genetic information can then be used as a tool for assessing the current conservation management plan for this species and for designing conservation strategies.

## Material and Methods

### Plant materials

**Figure 1:**
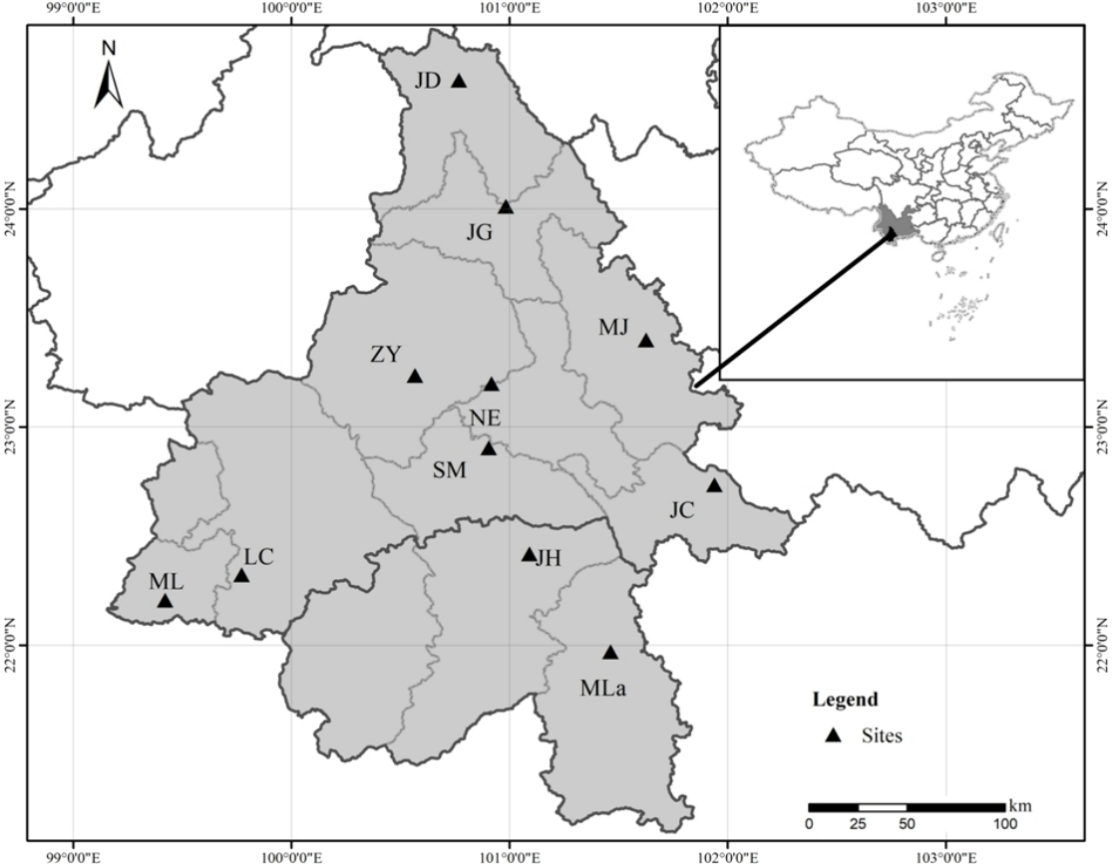
Geographical location of the 11 Natural populations of Simao pine in China.

Eleven natural populations of Simao pine with a total of 290 individuals were sampled throughout the species distribution range (Fig. 1 and Table 1). Field experiments were approved by Southwest Forestry University (project number: 2013Y121). In large populations (*n* >  100), 30 adult individuals were selected within a distance of >  100 m. In small populations (*n* <30), all available adult individuals were sampled. For each sampled individual, young fresh pine needles (∼10 g) were collected, dried in silica gel, and stored at −80 °C until subsequent DNA extraction.

**Table 1:** Locations and geographic characteristics of the sampled Simao pine populations.

Population name	Population abbreviation	Longitude (°E)	Latitude (°N)	Altitude (m)	T_mean_ (°C)	T_max_ (°C)	T_min_ (°C)	Pr (mm)	Pr_max_ (mm)	Pr_min_ (mm)	Number
Mojiang	MJ	101.62	23.40	1,578	19.0	28.2	5.9	279	1,322	15	30
Ninger	NE	100.91	23.19	1,383	20.1	29.7	6.4	283	1,342	13	30
Jingdong	JD	100.76	24.59	1,485	19.0	28.5	5.3	191	965	13	30
Jinggu	JG	100.98	24.01	1,525	19.8	29.5	5.8	285	1,342	13	30
Zhenyuan	ZY	100.58	23.23	1,964	16.8	25.9	3.3	238	1,144	14	30
Jiangcheng	JC	101.93	22.73	1,263	16.9	25.6	4.4	398	1,772	22	30
Lancang	LC	99.77	22.32	1,725	17.8	27.4	3.6	314	1,520	12	30
Menglian	ML	99.42	22.21	1,239	18.7	28.5	4.4	283	1,449	9	30
Jinghong	JH	101.09	22.41	870	20.6	30.2	7.6	290	1,434	15	20
Mengla	Mla	101.46	21.96	1,032	19.0	28.0	6.4	319	1,645	20	20
Simao	SM	100.90	22.90	1,355	18.4	27.9	4.3	324	1,491	14	10

### DNA extraction

Genomic DNA was isolated from the pine needles of each individual tree according to the cetyltrimethyl ammonium bromide (CTAB) method (Wang, Li & Li, 2011). All genomic DNA was kept frozen at −20 °C for standby application.

### SRAP-PCR Amplification Analysis

Fifteen primer combinations (Table 2), selected from the initial 100 pairs of primer combinations, were used for the study. Next, SRAP-PCRs were run in 25 µL volumes containing 60 ng DNA template, 2.0 mM MgCl_2_, primer 1 µM, dNTPs 0.2 mM, and 1U *Taq* DNA polymerase (Fermentas, Ottawa, Canada). The following cycling parameters were used for amplification: 4 min denaturing at 94 °C, five cycles of 94 °C for 1 min; 35 °C for 45 s and 72 °C for 1 min, 30 cycles of 94 °C for 1 min, annealing for 1 min, 72 °C for 1 min, and 5 min 72 °C for final extension. The PCR products were stored at −20 °C. Finally, amplification products were separated on 6% denaturing polyacrylamide gel and visualized by silver nitrate staining.

**Table 2:** Primer sequences used for SRAP analysis.

Primer combination	Sequences (3′–5′)	
Me1-Em4	TGAGTCCAAACCGGATA	GACTGCGTACGAATTTGA
Me2-Em9	TGAGTCCAAACCGGAGC	GACTGCGTACGAATTGAG
Me4-Em9	TGAGTCCAAACCGGACC	GACTGCGTACGAATTGAG
Me5-Em10	TGAGTCCAAACCGGAAG	GACTGCGTACGAATTGCC
Me6-Em2	TGAGTCCAAACCGGTAA	GACTGCGTACGAATTTGC
Me6-Em3	TGAGTCCAAACCGGTAA	GACTGCGTACGAATTGAC
Me6-Em5	TGAGTCCAAACCGGTAA	GACTGCGTACGAATTAAC
Me6-Em9	TGAGTCCAAACCGGTAA	GACTGCGTACGAATTGAG
Me7-Em1	TGAGTCCAAACCGGTCC	GACTGCGTACGAATTAAT
Me7-Em7	TGAGTCCAAACCGGTCC	GACTGCGTACGAATTCAA
Me8-Em4	TGAGTCCAAACCGGTGC	GACTGCGTACGAATTTGA
Me9-Em6	TGAGTCCAAACCGGAAC	GACTGCGTACGAATTGCA
Me9-Em7	TGAGTCCAAACCGGAAC	GACTGCGTACGAATTCAA
Me9-Em9	TGAGTCCAAACCGGAAC	GACTGCGTACGAATTGAG
Me10-Em3	TGAGTCCAAACCGGTAG	GACTGCGTACGAATTGAC

### Data statistics and analysis

The SRAP bands of all 15 pairs of primer combination were graded with presence (1) or absence (0) and transformed into a 1/0 binary character matrix. The binary data matrix was analyzed using the program POPGEN v.1.32 (Yeh, Yang & Boyle, 1999) to estimate genetic diversity parameters, including the percentage of polymorphic bands (*P*, %), Nei’s gene diversity analysis (*H*), Shannon’s information index (*I*), the observed number of alleles (*N*_*a*_), and the effective number of alleles (*N*_*e*_). Total gene diversity (*H*_*t*_), genetic diversity within populations (*H*_*s*_) as well as the relative magnitude of genetic differentiation among populations (*G*_*st*_), gene flow (*N*_*m*_), and Nei’s genetic distance were also evaluated. Polymorphic information content (PIC) was calculated using a simplified formula (Anderson et al., 1993).

Genetic differentiation within and among populations was estimated by the analysis molecular of variance (AMOVA) software package in GenAIEx 6.5 (Peakall & Smouse, 2006). To analyze the quality of information from particular SRAP primers, principal component analysis (PCA) was used by GenAIEx 6.5 (Peakall & Smouse, 2006). A dendrogram was generated using the unweighted pair group method with the arithmetic mean (UPGMA) clustering procedure of NTSYS-pc v. 2.02 (Rohlf, 2000), and the relationship between geographic and genetic distances was performed with the Mantel test by GenAIEx 6.5 (Peakall & Smouse, 2006). We further assessed the genetic structure of populations using the Bayesian clustering approach implemented in the software STRUCTURE v.2.3.3 (Pritchard, Stephens & Donnelly, 2000). The number of potential genetic clusters (*K* values) was run from 1 to 20, with 10 independent runs for each *K*. The contribution to the genotypes of the accessions was calculated based on 10^5^ iteration burn-in and 10^5^ iteration sampling periods. Then, the optimal number of clusters *K* was identified following the procedure of Evanno, Regnaut & Goudet (2005).

The ecological data, i.e., annual precipitation (Pr), maximum monthly precipitation (Pr_max_), minimum monthly precipitation (Pr_min_), annual mean temperature (T_mean_), maximum temperature of the warmest month (T_max_), and minimum temperature of the coldest month (T_min_) were extracted from Worldclim-Global Climate Data1 with ArcGIS 9.3 (Table 1, Hijmans et al., 2005). Correlations among ecological factors and genetic diversity parameters were determined using a Spearman nonparametric correlation coefficient matrix constructed with SPSS version 18.

## Results

### SRAP-PCR amplification

The selected 15 primer combinations were used to amplify 11 natural populations, and the statistics of amplified primer sites were calculated by the binary character coding method (Table 3). This method was used to amplify 132 sites in the range of 100–1,000 bp. Among these sites were 126 polymorphic loci, the percentage of polymorphism was as high as 95.45%, and each primer amplified an average of 8.8 loci and 8.4 polymorphic loci. This indicated that there was rich genetic diversity among Simao pine, and the genetic background was very complex. The number of polymorphic loci amplified by primer combination Me9-Em6 and Me9-Em7 was 12, which was the highest, and the number of polymorphic loci amplified by primer combination Me4-Em9, Me6-Em3, Me6-Em5, Me9-Em9 was 7, which was the least. In the 15 primer combinations, the percentage of polymorphic loci of primer combination Me6-Em9 and Me8-Em4 were the lowest, at 88%, Me7-Em1 and Me9-Em6 were 90% and 91.66% respectively, and the remaining primer combinations were all 100%. The PIC ranged from 0.43 to 0.77, with an average of 0.63.

**Table 3:** Analysis of SRAP-PCR amplification results.

Primer combinations	Total number of bands	PB	PPB%	PIC
M1-E4	9	9	100	0.61
M2-E9	9	9	100	0.72
M4-E9	7	7	100	0.64
M5-E10	10	8	80	0.43
M6-E2	8	8	100	0.69
M6-E3	7	7	100	0.65
M6-E5	7	7	100	0.65
M6-E9	9	8	88.88	0.63
M7-E1	10	9	90.00	0.51
M7-E7	8	8	100	0.56
M8-E4	9	8	88.88	0.45
M9-E6	12	11	91.66	0.77
M9-E7	12	12	100	0.76
M9-E9	7	7	100	0.70
M10-E3	8	8	100	0.62
Total	132	126		
Average	8.8	8.4	95.45	0.63

**Notes.**

(PB) Number of Polymorphic Bands (PPB%) Number of Percentage of polylnorphic bands (PIC) Polymorphic Information Content

### Genetic diversity analysis

At the population level, the percentage of polymorphic bands (*P*) ranged from 77.27% (A) to 100% (C), with an average of 93.46%, and 100.0% at species level. The mean observed number of alleles (*N*_*a*_) ranged from 1.7727 to 2.0000, while the mean effective number of alleles (*N*_*e*_) varied from 1.5469 to 1.8181. Nei’s genetic diversity (*H*) varied from 0.3089 to 0.4418, with an average of 0.3801, and Shannon’s information indices (*I*) ranged from 0.4511 to 0.6310, with an average of 0.5529. At species level, *H* and *I* were 0.4567 and 0.6484, respectively (Table 4).

**Table 4:** Genetic diversity of 11 Simao pine populations.

Pop	*N*_*a*_	*N*_*e*_	*P*	*H*	*I*	*H*_*t*_	*H*_*s*_	*G*_*st*_	*N*_*m*_
SM	1.7727	1.5469	77.27	0.3089	0.4511				
NE	1.9394	1.6889	93.94	0.3862	0.5611				
MJ	1.9470	1.7274	94.70	0.4019	0.5799				
JD	1.9394	1.6818	93.94	0.3819	0.5555				
JG	1.9318	1.6759	93.18	0.3781	0.5498				
ZY	1.9091	1.6480	90.91	0.3627	0.5288				
JC	2.0000	1.8181	100.00	0.4418	0.6310				
LC	1.9545	1.7072	95.45	0.3965	0.5754				
ML	1.9545	1.6384	95.45	0.3687	0.5430				
JH	1.9773	1.7005	97.73	0.3945	0.5750				
Mla	1.9545	1.6259	95.45	0.3600	0.5313				
Mean	1.9346	1.6781	93.46	0.3801	0.5529				
Total	2.0000	1.8516	100.00	0.4567	0.6484	0.4580	0.3801	0.1701	2.4403

**Notes.**

*N*_*a*_ observed number of alleles *N*_*e*_ effective number of alleles *P* percentage of polymorphic loci *H* Nei’s gene diversity *I* Shannon’s information indices *H*_*t*_ total genetic diversity *H*_*s*_ genetic diversity within populations *G*_*st*_ the relative magnitude of genetic differentiation among populations *N*_*m*_ gene flow among populations

### Genetic differentiation

The total genetic diversity (*H*_*t*_) of the species and genetic diversity within populations (*H*_*s*_) were 0.4580 and 0.3801, respectively (Table 4). The proportion of genetic variation contributed by differences among populations (*G*_*st*_) was 0.1701, thus leaving 82.99% of the total genetic variation kept within the populations. The average *N*_*m*_ obtained was 2.4403, suggesting the existence of a certain degree of gene flow among natural distribution populations. This finding was consistent with the results of AMOVA, which detected the highest genetic variation within populations (86%), whereas the variance among populations was only 14% (Table 5).

**Table 5:** Analyses of molecular variance (AMOVA) for Simao pine by SRAP.

Source of variation	*d*.*f*.	Sum of squares	Variance compent	Percentage of variation%	*P* value
Among Pops	10	1,343.086	4.146	14%	0.139
Within Pops	279	7,160.500	25.665	86%	0.010
Total	289	8,503.586	29.810	100%	

### Cluster analysis

Based on the SRAP data, a broad range of Nei’s genetic distance was found to exist among the 11 Simao pine natural populations, varying from 0.0722 to 0.2820 (Table 6), with a mean of 0.1483. The highest genetic distance pairs were found between population SM and MLa (0.2820), and these populations may be good sources for further breeding purposes. The lowest genetic distance pairs were found between populations JD and ZY (0.0722).

**Table 6:** Nei’s genetic distances and geographical distances among 11 populations.

	SM	NE	MJ	JD	JG	ZY	JC	LC	ML	JH	Mla
SM	****	32.76	92.15	187.44	50.61	123.16	107.94	133.22	171.17	57.27	118.53
NE	0.0826	****	75.92	155.03	36.23	90.48	116.85	152.635	189.02	88.41	147.32
MJ	0.1743	0.0788	****	158.33	109.97	94.52	80.35	224.71	262.28	121.92	159.41
JD	0.2025	0.1214	0.0726	****	151.42	97.75	237.96	271.14	297.85	243.03	299.15
JG	0.2135	0.1499	0.124	0.0661	****	95.92	151.44	130.17	163.98	105.53	168.09
ZY	0.2178	0.1296	0.0861	0.0722	0.0741	****	172.16	224.62	256.23	177.05	231.72
JC	0.1855	0.1135	0.0943	0.0927	0.0948	0.082	****	227.86	265.85	94.26	98.16
LC	0.2529	0.1662	0.1883	0.1969	0.1672	0.1808	0.099	****	38.31	136.48	178.92
ML	0.2433	0.1538	0.1666	0.1835	0.1694	0.1830	0.1142	0.0649	****	173.71	212.45
JH	0.2511	0.1428	0.1228	0.1605	0.1356	0.1480	0.0879	0.0747	0.0769	****	62.84
Mla	0.2820	0.1796	0.1959	0.1993	0.1899	0.2265	0.1299	0.1633	0.1924	0.1433	****

**Notes.**

Above diagonal: geographical distances (km); below diagonal: genetic distances.

**Figure 2:**
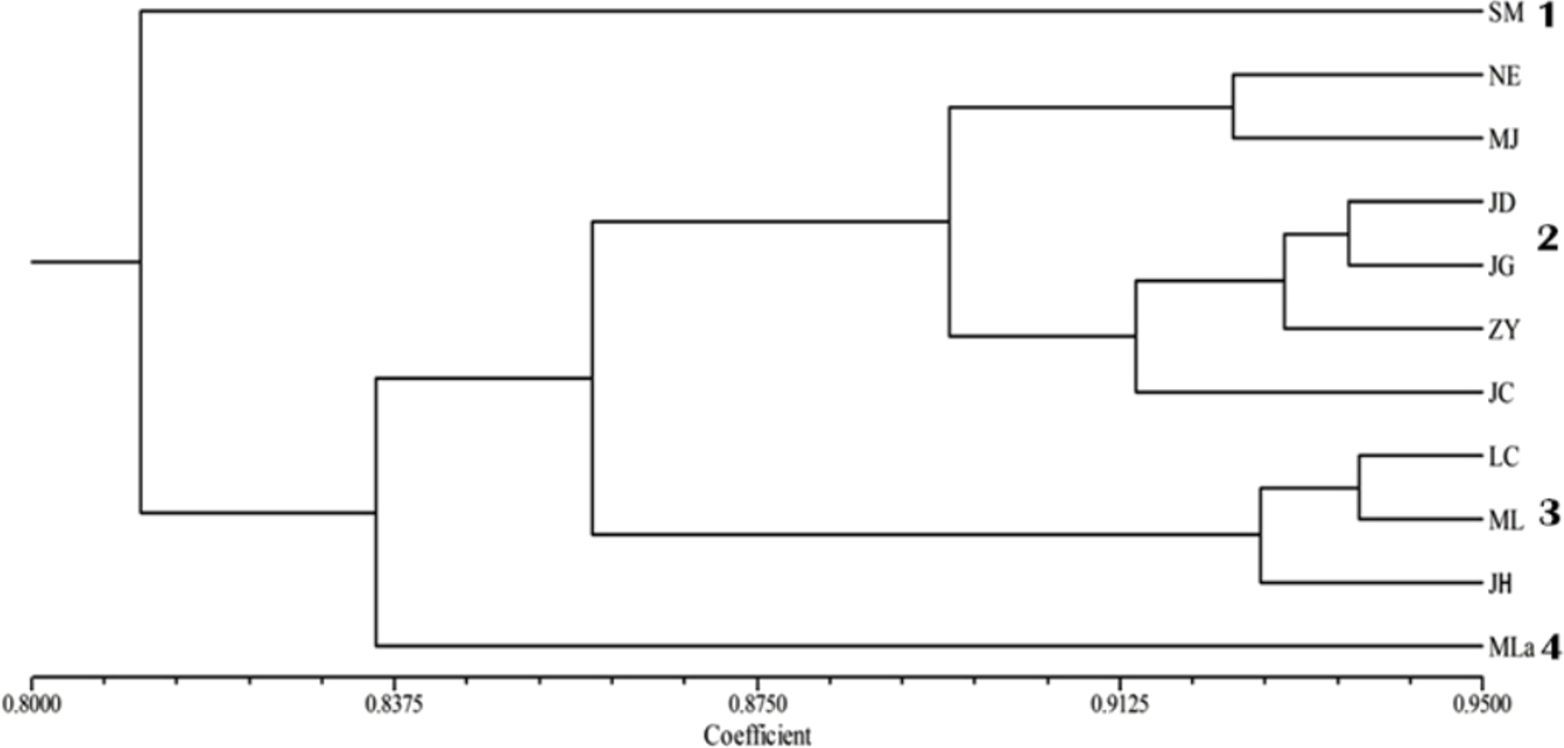
UPGMA cluster analysis of genetic similarity of 11 populations.

The applied measure of genetic similarity was used to construct UPGMA dendrograms (Fig. 2). The 11 populations were divided into four groups with population SM forming one subgroup; Mla forming another; the populations NE, MJ, JD, JG, ZY and JC forming the third group; and LC, ML and JH were included in the fourth group. The clustering results were not specified completely from location. Furthermore, the dendrogram showed that the populations were partly mixed clusters, although geographically more distant (Fig. 2). Consistent with these results, the Mantel test revealed that there was no significant correlation between geographic and genetic distance (*r* = 0.241, *p* = 0.090).

### Principal coordinate analysis

**Figure 3:**
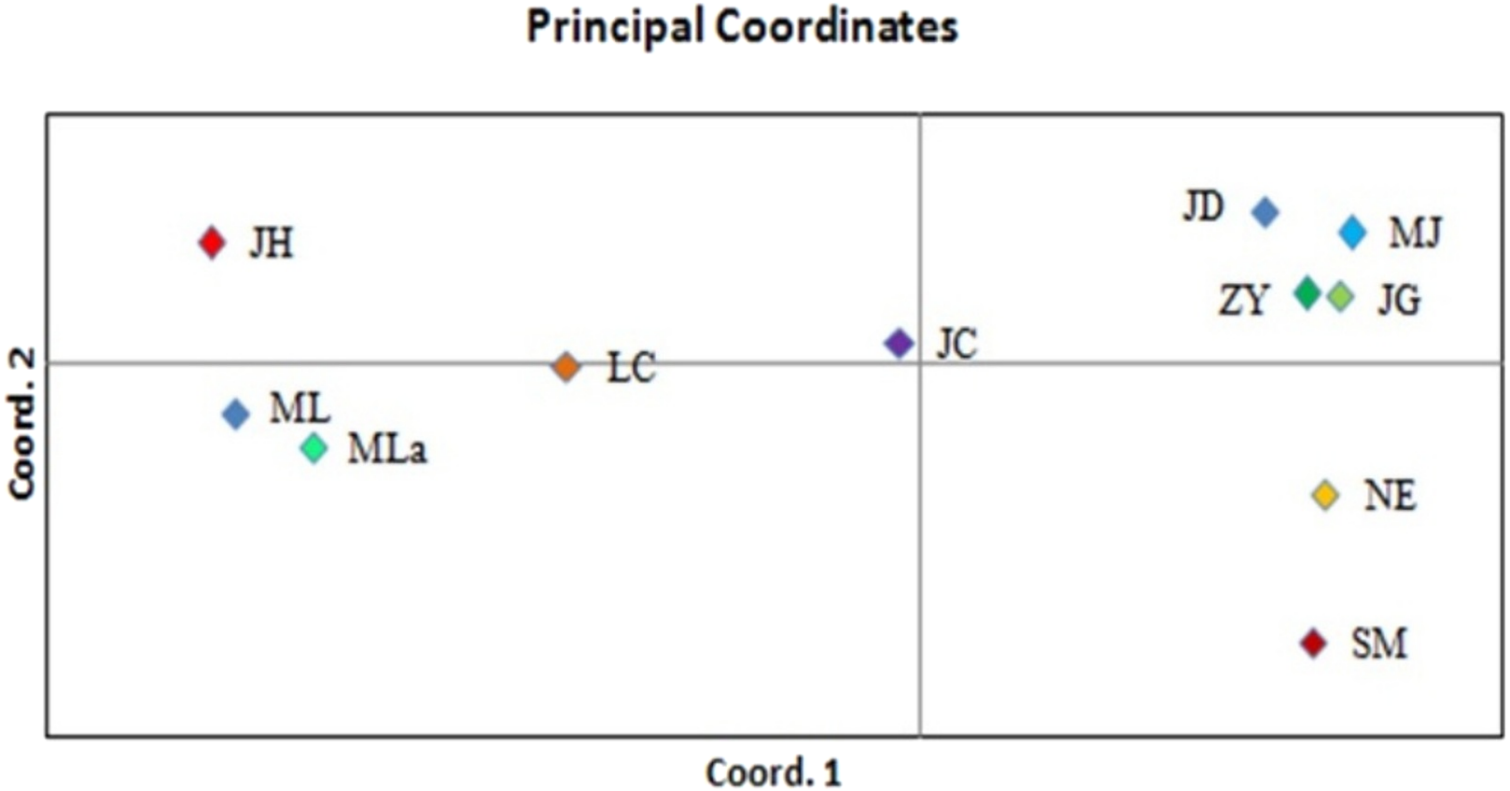
PCoA of 11 populations based on variability.

Principal coordinate analysis (PCoA) was performed to determine the genetic relationships among the accessions with minimum distortion. The first two principal components displayed 37.69% and 60.12% of total variation, respectively, and the 72.74% was expressed by the first three components (Fig. 3). Corresponding with the cluster analysis, each population formed a separate plot and could be clearly distinguished from those of other populations. The first principal coordinate separated 11 populations into two groups. The first group was composed of six high latitude populations including JD, MJ, ZY, JG, NE and SM, and the remaining five southern populations were separated into the second group. Based on the results of the first principal coordinate, the second principal coordinate separated 11 populations into four groups.

### Population structure analysis

**Figure 4:**
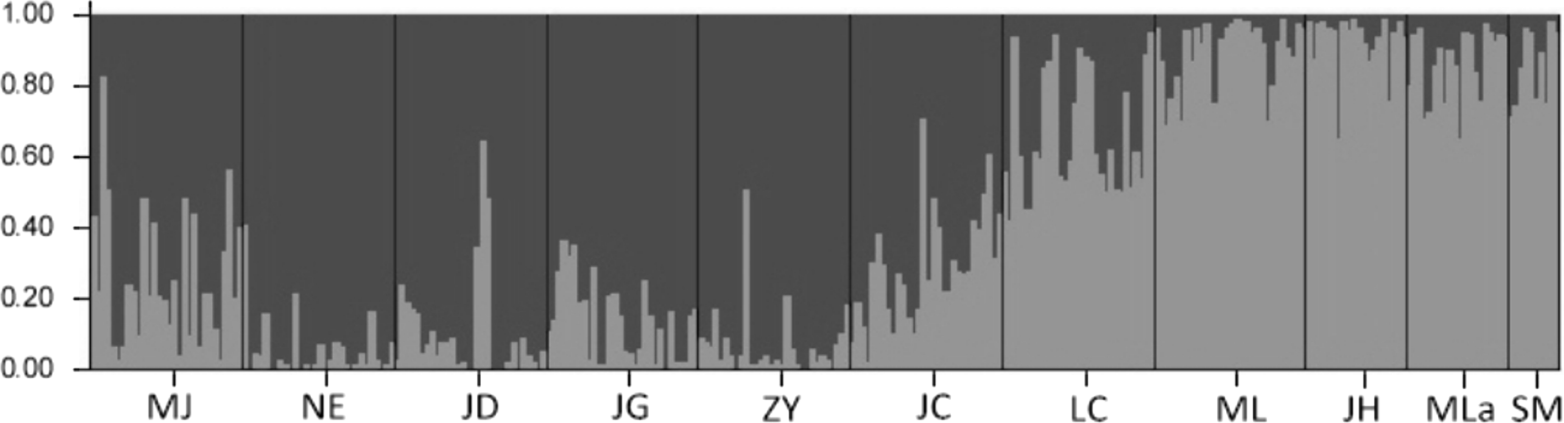
The proportions of cluster memberships at the individual level in 11 Simao pine populations in the two clusters identified by STRUCTURE.

The results of the Bayesian clustering analysis of genetic structure showed that the populations analyzed of Simao pine, best fit two genetic groups (*K* = 2, Fig. 4). The percentages of individuals in cluster I and cluster II were 61.87 and 30.38% respectively, whereas 7.75% of the individuals had mixed membership, with *Q* scores of 0.2 < *Q* < 0.8. The individuals in NE, JD, JG, ZY populations with *Q* values of >0.8 belonged to cluster I, and the ML, JH, Mla, SM populations with *Q* values of <0.2 belonged to cluster II. Individuals with admixed population assignments were from MJ, JC and LC populations.

### Correlation among ecological factors and genetic diversity parameters

Based on the ecological factors and genetic diversity parameters data, a Spearman nonparametric correlation coefficient matrix had been constructed by SPSS. The result showed no significant correlations between the genetic diversity parameters and geoclimatic variables.

## Discussion

In this study, SRAP markers were used to evaluate levels of genetic variation within and among 11 natural populations of Simao pine in China. Previously, three natural populations of Simao pine genetic variation were investigated using nine enzyme systems (Chen, Zhao & Wang, 2002). The reported values of the proportion of polymorphic loci, mean genetic distance, and genetic differentiation were all considerably lower than the results of this study. These differences were likely due to the ability of SRAP markers to detect more loci and higher levels of polymorphism than isozymes markers. One of the benefits of SRAP over other molecular techniques was its sensitivity in stock identification without any upfront knowledge of the species genome, which provided a large number of independent markers that can be rapidly analyzed (Uzun et al., 2009; Soleimani, Talebi & Sayed-Tabatabaei, 2012; Ahamd et al., 2014; Peng et al., 2015).

The PIC and polymorphism rate (*P*) were used to measure genetic diversity. A high level of polymorphism was observed in numerous amplification products generated in the course of the analysis. In this study, PIC ranged from 0.43 to 0.77, with an average of 0.63, indicating that the SRAP markers could develop high loci polymorphism useful for genetic variation of accessions studied in this research (Talebi, Rahimmalek & Norouzi, 2015). The percentage of polymorphic bands (*P*) of Simao pine was 93.46%, revealing a considerable level of genetic diversity, similar to that of its relatives *Pinus yunnanensis* (*P* = 96.43%; Xu et al., 2015). At a species level, Nei’s genetic diversity (*H*) was 0.4567 and Shannon’s information indices (*I*) was 0.6484, indicating that Simao pine had a high basis of genetic diversity. Previous studies also showed that narrowly distributed species could have higher genetic diversity (Dolan et al., 1999; Wang et al., 2013).

In the present study, the total genetic diversity (*H*_*t*_) of the species and genetic diversity within populations (*H*_*s*_ ) were estimated to be 0.4580 and 0.3801 respectively. Compared to previous SRAP-based studies of pine plants, Simao pine showed higher diversity than *Pinus taxa* (*H*_*t*_ = 0.2134; *H*_*s*_ = 0.3426; (Xie et al., 2015), but lower genetic diversity than khasi pine (*H*_*t*_ = 0.547; *H*_*s*_ = 0.285; (Rai, Ginwal & Saha, 2017). Genetic differentiation (*G*_*st*_) was the ratio between the additive genetic variance among populations and the total additive genetic variance (Wright, 1965). The study revealed low genetic differentiation (*G*_*st*_ = 0.1701) among populations of Simao pine. This value was higher than the mean value (*G*_*st*_ = 0.073) recorded for 121 woody species examined using allozyme markers (Hamrick, Godt & Sherman-Broyles, 1992). The low gene differentiation among populations was mainly caused by the high level of gene flow, gene flow could give play to its homogenizing function to prevent differentiation among each population caused by genetic drift. Similar results have been reported for several woody trees that have high levels of genetic diversity, and even in trees with low or moderate levels of genetic differentiation in populations that were located several kilometers away (Lacerda et al., 2001; Arias et al., 2012). The AMOVA analysis revealed that 86% of the variation was within populations, while the remaining 14% was among populations. This was in agreement with a number of studies where it has been observed that conifers showed high levels of genetic variation within populations and relatively little differentiation among populations (Hiebert & Hamrick, 1983; Agundez et al., 1997; Xie et al., 2015).

Gene flow is the transfer of alleles from one population to another and the study of this is critical for understanding the evolution of plants and population process within and among species (Grant, 1991; Gerber et al., 2014). Gene flow among populations is mainly produced by foreign genes brought by pollens and seeds for spermatophytes (Hamrick, 1987). The *N*_*m*_ of Simao pine was 2.4403, showed that gene exchange among populations was moderately frequent, and weakens genetic differentiation among populations. Gene exchange in the present study was enough to rule out the possibility that some differentiation among the populations of Simao pine could be due to isolation. This result was consistent with the lack of correlation between genetic and geographic distances (*r* = 0.241, *p* = 0.090), which suggested no significant geographic restriction to gene flow among the populations. The reason for slightly higher *N*_*m*_ may be due to the morphological character of Simao pine, as tall plants benefit from reduced resistance to pollen movement in the air. A long-distance pollen flow also enhanced the gene recombination (Feng et al., 2009). Furthermore, a lack of effective geographic isolation may also have contributed to the improvement of gene homogenization among provenances. Simao pine forests were mainly distributed in low mountainous upland in southern China, with a closer distance between provenances.

The genetic relationship among the 11 analyzed populations of Simao pine was represented in a dendrogram showing a clear genetic differentiation of the SM population, which was genetically distinct and could be considered to be more genetically diverse compared to the other ten populations. Principal coordinate analysis (PCoA) was performed to provide spatial representation of the relative genetic distances among populations defined by cluster analysis. The results of UPGMA and PCoA also showed that the assignment of populations was not completely consistent with their geographic origin, a result that has been observed in previous studies (Liu et al., 2013; Cheng, Zheng & Sun, 2015). This finding indicated that geographical distance had no obvious effect on genetic differentiation of Simao pine populations.

A Bayesian cluster analysis performed with STRUCTURE, showed that the most probable number of genetic groups in the data was two (*K* = 2) for Simao pine. Cluster I was composed of the NE, JD, and ZY populations from the northwest of the Simao pine natural range, collected from regions that had higher altitude (mostly above 1,400 m) and latitude. The ML, JH, Mla, and SM populations which belonged to cluster II were collected from southern regions that had lower altitude (mostly below 1,300 m) and latitude. These results possibly reflected an adaptational difference. It implied that geographical and environmental factors together created stronger and more discrete genetic differentiation than isolation by distance alone.

In the study, there were no significant correlations between the ecological factors and genetic diversity parameters according to Spearman nonparametric correlation analysis. It was suggested that the ecological factors had less effect on genetic diversity. This may be due to the limited distribution, and the small geographical and ecological differences of the studied populations. The high level of genetic diversity in Simao pine is mainly caused by the traits of long-life, generation overlap, wind pollination, and wind-seed dispersal, which lay a broad genetic base for the species (Chen, Zhao & Wang, 2002).

Retaining genetic diversity was the key to multi-generation improvement of forest trees and fine provenances should have an extensive genetic base and significant genetic gain (Feng et al., 2009). Thorough understanding of the extent and patterns of genetic diversity in Simao pine was essential for its conservation and utilization. The results reported here revealed high genetic diversity at the population level and low genetic diversity at the species level in Simao pine. Neither the percentage of polymorphic loci nor diversity index displayed significant difference among the 11 provenances. Therefore, selective breeding of Simao pine forests can disregard the limitation of geographic location and should directly select individuals with advanced growth traits. The low genetic differentiation among populations implied that conservation efforts should aim to preserve all existing populations of this species. Based on this, the in situ conservation method was proposed as it is of paramount urgency that sufficient natural population numbers and sizes were conserved to prevent a reduction in genetic diversity. Moreover, in order to achieve effective conservation of germplasm resources, efforts were required to carefully plan and construct pollen and gene banks for Simao pine. In the meantime, natural protection areas should be established to conserve and restore the habitat and populations. In this study, population JC displayed relatively high genetic diversity, and should therefore be a priority site for in situ conservation. In addition, it was important to develop a core collection of Simao pine, which would not only mitigated the pressure of excessive exploitation of wild resources, but can also helped in achieving more effective management and utilization of germplasm.

## Conclusions

The present study is understood to be the first genetic investigation of Simao pine using SRAP markers to understand distribution and genetic variation. The results indicated that SRAP markers can be efficiently used in the study of genetic diversity and genetic variability of Simao pine. The study revealed high levels of genetic diversity and low or moderate levels of genetic differentiation. Slightly frequent gene flow existed within Simao pine populations, weakening genetic differentiation among these populations. Based on these results, conservation strategies for Simao pine have been formulated.

##  Supplemental Information

10.7717/peerj.6529/supp-1 Data S1 SRAP binary character matrix

10.7717/peerj.6529/supp-2 Data S1 Analysis of POPGENand GenAIEx

10.7717/peerj.6529/supp-3 Data S1 Correlation among ecological factors and Genetic Diversity matrix

10.7717/peerj.6529/supp-4 Supplemental Information 1 The uncropped gel pictures of m2Xe9, m4Xe9, m5Xe10, m6Xe5 primer combination

10.7717/peerj.6529/supp-5 Supplemental Information 2 The uncropped gel pictures of m6Xe9, m6Xe2, m6Xe3, m7Xe1 primer combination

10.7717/peerj.6529/supp-6 Supplemental Information 3 The uncropped gel pictures of m7Xe7, m8Xe4, m9Xe6 primer combination

10.7717/peerj.6529/supp-7 Supplemental Information 4 The uncropped gel pictures of m9Xe7, m9Xe9, m10Xe3 primer combination

## Additional Information and Declarations

### Field Study Permissions

The following information was supplied relating to field study approvals (i.e., approving body and any reference numbers): Field experiments were approved by the Southwest forestry university (project number: 2013Y121).

### Data Availability

The following information was supplied regarding data availability: The raw data are available in the Supplemental Files.

## References

[ref-1] Agundez D, Degen B, Von Wuehlisch G, Alia R (1997). Genetic variation of Aleppo pine (*Pinus halepensis* MILL.) in Spain. Forest Genetics.

[ref-2] Ahamd R, Farhatullah QCF, Rahman H, Swati ZA (2014). Genetic diversity analyses of *Brassica Napus* accessions using SRAP molecular markers. Plant Genetic Resources.

[ref-3] Anderson JA, Churchill GA, Autrique JE, Tanksley SD, Sorrells ME (1993). Optimizing parental selection for genetic linkage maps. Genome.

[ref-4] Arias DM, Albarran L, González-Rodríguez A, Penaloza RJ, Oscar D, Leyva E (2012). Genetic diversity and structure of wild populations of the tropical dry forest tree *Jacaratia Mexicana* (Brassicales: Caricaceae) at a local scale in Mexico. Revista de Biología Tropical.

[ref-5] Bhatt J, Kumar S, Patel S, Solanki R (2017). Sequence-related amplified polymorphism (SRAP) markers based genetic diversity analysis of cumin genotypes. Annals of Agrarian Science.

[ref-6] Cai NH, Xu YL, Wang DW, Chen S, GenQian L (2017). Identification and characterization of microsatellite markers in *pinus kesiya* var. *langbianensis* (pinaceae). Applications in Plant Sciences.

[ref-7] Chen SY, Zhao WS, Wang J (2002). Genetic diversity and genetic differentiation of natural populations of *pinus kesiya* var. *langbinanensis*. Journal of Forestry Research.

[ref-8] Cheng BB, Zheng YQ, Sun QW (2015). Genetic diversity and population structure of *taxus cuspidata* in the changbai mountains assessed by chloroplast dna sequences and microsatellite markers. Biochemical Systematics & Ecology.

[ref-9] Dolan RW, Yahr R, Menges ES, Halfhill MD (1999). Conservation implications of genetic variation in three rare species endemic to Florida Rosemary Scrub. American Journal of Botany.

[ref-10] Evanno G, Regnaut S, Goudet J (2005). Detecting the number of clusters of individuals using the software STRUCTURE: a simulation study. Molecular Ecology.

[ref-11] Feng FJ, Chen MM, Zhang DD, Xin S, Han SJ (2009). Application of srap in the genetic diversity of *pinus koraiensis* of different provenances. African Journal of Biotechnology.

[ref-12] Gerber S, Chadoeuf J, Gugerli F, Lascoux M, Buiteveld J, Cottrell J, Dounavi A, Fineschi S, Forrest LL, Fogelqvist J, Goicoechea PG, Jensen JS, Salvini D, Vendramin GG, Kremer A (2014). High rates of gene flow by pollen and seed in Oak populations across Europe. PLOS ONE.

[ref-13] Grant V (1991). The evolutionary process: a critical study of evolutionary theory.

[ref-14] Hamrick JL, Urbanska K (1987). Gene flow distributing of genetic variation in plant populations. Differentiation patterns in higher plants.

[ref-15] Hamrick JL, Godt MJW, Sherman-Broyles SL (1992). Factors influencing levels of genetic diversity in woody plant species. New Forests.

[ref-16] Hiebert RD, Hamrick JL (1983). Patterns and levels of genetic variation in Great Basin bristlecone pine, *Pinus longaevea*. Evolution.

[ref-17] Hijmans RJ, Cameron SE, Parra JL, Jones PG, Jarvis A (2005). Very high resolution interpolated climate surfaces for global land areas. International Journal of Climatology.

[ref-18] Lacerda DR, Acedo MD, Filho JP, Lovato MB (2001). Genetic diversity and structure of natural populations of *plathymenia reticulata*, (mimosoideae), a tropical tree from the Brazilian cerrado. Molecular Ecology.

[ref-19] Li SF, Lang XD, Liu WD, Ou GL, Xu H, Su JR (2018b). The relationship between species richness and aboveground biomass in a primary *Pinus kesiya* forest of Yunnan, southwestern China. PLOS ONE.

[ref-20] Li G, Quiros CF (2001). Sequence-related amplified polymorphism (SRAP), a new marker system based on a simple PCR reaction: its application to mapping and gene tagging in Brassica. Theoretical and Applied Genetics.

[ref-21] Li BJ, Wang JY, Liu ZJ, Zhuang XY, Huang JX (2018a). Genetic diversity and ex situ conservation of *Loropetalum subcordatum*, an endangered species endemic to China. BMC Genetics.

[ref-22] Liu L, Chen W, Zheng X, Li J, Yan DT, Liu L, Liu X, Wang YL (2016). Genetic diversity of *Ulmus lamellosa* by morphological traits and sequence-related amplified polymorphism (SRAP) markers. Biochemical Systematics and Ecology.

[ref-23] Liu JF, Shi SQ, Chang E, Yang WJ, Jiang ZP (2013). Genetic diversity of the critically endangered *thujas utchuenensis* revealed by issr markers and the implications for conservation. International Journal of Molecular Sciences.

[ref-24] Ma Y, Qu SQ, Xu XR, Liang TT, Zang DK (2015). Genetic diversity analysis of Cotoneaster schantungensis Klotz. Using SRAP marker. American Journal of Plant Sciences.

[ref-25] Ou GL, Wang JF, Xu H, Chen KY, Zheng HM, Zhang B, Sun XL, Xu TT, Xiao YF (2016). Incorporating topographic factors in nonlinear mixed-effects models for aboveground biomass of natural Simao pine in Yunnan, China. Journal of Forestry Research.

[ref-26] Peakall R, Smouse PE (2006). GENALEX 6: genetic analysis in excel, population genetic software for teaching and research. Molecular Ecology Notes.

[ref-27] Peng X, Ji QY, Fan SW, Zhang YJ, Zhang JJ (2015). Genetic diversity in populations of the endangered medicinal plant *Tetrastigma hemsleyanum* revealed by ISSR and SRAP markers: implications for conservation. Genetic Resources and Crop Evolution.

[ref-28] Pritchard JK, Stephens M, Donnelly P (2000). Inference of population structure using multilocus genotypes data. Genetics.

[ref-29] Rai KC, Ginwal HS, Saha R (2017). Genetic diversity assessment of Khasi pine (*pinus kesiya* Royle ex. Gordon) from Meghalaya using chloroplast microsatellite markers. Indian Journal of Forestry.

[ref-30] Rohlf FJ (2000).

[ref-31] Shaye NA, Migdadi H, Charbaji A, Alsayegh S, Daoud S, Al-Anazi W, Alghamdi S (2018). Genetic variation among saudi tomato (*solanum lycopersicum* L.) landraces studied using sds-page and srap markers. Saudi Journal of Biological Sciences.

[ref-32] Soleimani MH, Talebi M, Sayed-Tabatabaei BE (2012). Use of SRAP markers to assess genetic diversity and population structure of wild, cultivated, and ornamental pomegranates (*Punica granatum* L.) in different regions of Iran. Plant Systematics and Evolution.

[ref-33] Talebi M, Rahimmalek M, Norouzi M (2015). Genetic diversity of Thymus daenensis subsp. daenensis using SRAP markers. Biologia.

[ref-34] Uzun A, Yesiloglu T, Aka-Kacar Y, Tuzcu O, Gulsen O (2009). Genetic diversity and relationships within *citrus* and related genera based on sequence related amplified polymorphism markers (SRAPs). Scientia Horticulturae.

[ref-35] Wang XM, Hou XQ, Zhang YQ, Yang R, Feng SF, Li Y, Ren Y (2012b). Genetic diversity of the endemic and medicinally important plant *Rheum officinale* as revealed by inter-simpe sequence repeat (ISSR) markers. International Journal of Molecular Science.

[ref-36] Wang HF, Lencinas MV, Friedman CR, Zhu ZX, Qiu JX (2012a). Understory plant diversity assessment of Szemao pine (*Pinus kesiya* var. *langbianensis*) plantations in Yunnan, China. Collectanea Botanica.

[ref-37] Wang DW, Li Y, Li ZQ (2011). Identification of a malespecific amplified fragment length polymorphism (aflp) and a sequence characterized amplified region (SCAR) marker in *Eucommia ulmoides* Oliv. International Journal of Molecular Sciences.

[ref-38] Wang BS, Mao JF, Zhao W, Wang XR (2013). Impact of geography and climate on the genetic differentiation of the subtropical pine *Pinus yunnanensis*. PLOS ONE.

[ref-39] Wang Y, Yuan XL, Hua M, Li J, Wang J (2018). Transcriptome and gene expression analysis revealed mechanisms for producing high oleoresin yields from Simao pine (*Pinus kesiya* var.*langbianensis*). Plant Omics Journal.

[ref-40] Wright S (1965). The interpretation of population structure by F-statistics with special regard to systems of mating. Evolution.

[ref-41] Xie Q, Liu ZH, Wang SH, Li ZQ (2015). Genetic diversity and phylogenetic relationships among five endemic *pinus* taxa (pinaceae) of china as revealed by srap markers. Biochemical Systematics and Ecology.

[ref-42] Xu YL, Cai NH, Woeste K, Kang XY, He CZ, Li GQ, Chen S, Duan AA (2015). Genetic diversity and population structure of *pinus yunnanensis* by simple sequence repeat markers. Forest Science.

[ref-43] Yeh FC, Yang RC, Boyle T (1999).

[ref-44] Zhao N, Shi R, Li B, Xiong Z, Wang J (2016). Correlation between indexes of resin tapping and chemical components of *Pinus kesiya* var.*langbianensis*. Journal of West China Forestry Science.

[ref-45] Zhu YF, Chen SY, Hao JB, Wu T (2017). Genetic analysis of superior clones and identification of specific marker of elite clones of *Pinus kesiya*. Agricultural Science and Technology.

